# Knowledge, attitude, and quality of life among newly diagnosed type 2 diabetic patients attending diabetic clinics at Bugando Medical Centre, Mwanza, Tanzania

**DOI:** 10.3389/fcdhc.2025.1634244

**Published:** 2025-09-26

**Authors:** Allen Rweyendera, Greyson Gwahula, Faraja Alexander, Yacinter Vedastus, Raymond Maziku, Monica Mukama, Edwin Silas, Illuminata Kafumu, Alphonce Ngerecha, Ally Tuwa, Peter Chilipweli, Hyasinta Jaka, Samuel Kalluvya

**Affiliations:** ^1^ School of Medicine, Catholic University of Health and Allied Sciences (CUHAS), Mwanza, Tanzania; ^2^ Development of Research and Consultancy, Community Health and Development Foundation (CHADF), Mwanza, Tanzania; ^3^ Department of Internal Medicine, Bugando Medical Center and Catholic University of Health and Allied Sciences (CUHAS), Mwanza, Tanzania; ^4^ Department of Public Health, Mwanza College of Health and Allied Sciences (MWACHAS), Mwanza, Tanzania; ^5^ Department of Community Medicine, School of Public Health, Catholic University of Health and Allied Sciences (CUHAS), Mwanza, Tanzania

**Keywords:** Type 2 diabetic, quality of life, diabetic clinics, Bugando Medical Centre (BMC), Mwanza, Tanzania

## Abstract

**Background:**

Diabetes mellitus, particularly type 2 diabetes, is a rapidly escalating global health issue. The World Health Organization projects a significant increase in diabetes prevalence worldwide, especially in developing countries. Various studies have explored the prevalence and impact of type 2 diabetes, revealing significant geographical disparities in the incidence and management of the disease. However, the extent to which knowledge and attitude influence newly diagnosed patients, particularly in low-resource settings like Mwanza, Tanzania, remains underexplored. Thus, the aim of this study was to assess the knowledge, attitude, and quality of life among newly diagnosed type 2 diabetic patients attending diabetic clinics at Bugando Medical Centre (BMC) in Mwanza, Tanzania.

**Methods:**

A cross-sectional survey was conducted among newly diagnosed type 2 diabetic patients attending BMC diabetic clinics from September 2024 to November 2024. Data were collected using a structured questionnaire that includes validated instruments such as the Diabetes Knowledge Test (DKT), the Diabetes Attitude Scale (DAS), and the WHOQol for measuring quality of life (QoL). The questionnaire captured demographic and clinical characteristics data, diabetes knowledge, attitudes toward the disease, and QoL indicators. Statistical analysis was performed to identify correlations between knowledge, attitude, and QoL.

**Results:**

This study involved 150 newly diagnosed type 2 diabetic patients at Bugando Medical Centre. The median age was 62 years (IQR 57–68), with 63.3% female patients and 92% married. Most participants had primary education (49.7%) and resided in urban areas (82%). Clinically, 66% had hypertension, and the median BMI was 28.4 kg/m², indicating overweight/obesity. The median HbA1c level was 7.4% (IQR 6.9–8.8). In terms of knowledge, the median score was 9 (IQR 7–10), with 69.3% having moderate knowledge, 29.3% high knowledge, and 1.3% low knowledge. Education level influenced knowledge, with 78.4% of primary-educated patients having moderate knowledge, while 42.6% of those with secondary education had high knowledge. Regarding attitude, 54.9% exhibited a negative attitude, with 61.3% feeling inferior due to diabetes and 64% struggling with daily disease management. However, 50% felt things were going well, and 48% believed diabetes had minimal impact on their lives. QoL varied across domains: the physical health mean score was 3.1 (SD ± 0.56), psychological 3.2 (SD ± 0.61), social 3.7 (IQR 2.7–3.7), and environmental 2.99 (SD ± 0.53). The overall QoL median score was 3.2 (IQR 2.8–3.5), indicating average wellbeing, with challenges in the environmental domain requiring targeted interventions.

**Conclusion:**

This study highlights the significant challenges faced by newly diagnosed type 2 diabetic patients at Bugando Medical Centre, including knowledge gaps, negative attitudes, and poor quality of life, particularly in the physical and environmental domains. The findings emphasize the need for comprehensive educational initiatives and psychological support to enhance self-management. Targeted interventions, especially for vulnerable groups like female patients, along with a multidisciplinary care approach, can improve diabetes management and overall wellbeing.

## 1 Introduction

Diabetes mellitus (DM) is a heterogeneous metabolic disorder characterized by chronic hyperglycemia with alterations of carbohydrate, fat, and protein metabolism (1). The disease is clinically categorized into two types: type 1 diabetes mellitus (T1DM) and type 2 diabetes mellitus (T2DM), depending on the age of the individual at onset and pathophysiological mechanism for diabetes occurrence. T2DM, which affects adults, represents approximately 98% of all cases of DM diagnosed globally; nevertheless, this proportion differs considerably among countries. According to the International Diabetes Federation (IDF) report of the year 2021, the world incidence of T2DM among adults was 536.6 million people (10.5%), and the report projected that there would be 783.2 million people (12.2%) living with diabetes worldwide by 2045 (2). DM is responsible for approximately 1.5 million deaths each year. The incidence of T2DM of 4.7% in the sub-Saharan Africa (SSA) region is quite low, although this incidence varies by country, with the highest number of people with T2DM living in more affluent countries (3). Tanzania is one of the countries in the SSA region with the highest prevalence of T2DM, and recently, the prevalence has been documented to be 7.8% (4). According to the World Bank collection of development indicators and the IDF, in 2021, approximately 2.9 million Tanzanians (10.3%) lived with DM. The prevalence of DM in the adult population of Mwanza was found to be 11.9% (5).

Diabetes mellitus, particularly type 2 diabetes, has become a significant global health concern, with the World Health Organization (WHO) predicting a substantial increase in diabetes prevalence over the coming decades. This chronic disease is associated with severe complications, including cardiovascular disease, neuropathy, retinopathy, and nephropathy, which significantly impact patients’ quality of life (QoL) and pose a considerable burden on healthcare systems worldwide (6). The growing prevalence of diabetes necessitates urgent attention to effective management strategies that include patient education and lifestyle modification to mitigate these complications (7, 8).

Quality of life in diabetic patients is intricately linked to their glycemic control and the presence of diabetes-related complications. Studies have demonstrated that patients with better knowledge of diabetes management tend to have better glycemic control and, consequently, a higher QoL (9). The Diabetes Knowledge Test (DKT) and other validated tools have been instrumental in assessing patients’ understanding of diabetes, which is crucial for developing effective self-management practices (10). Moreover, a positive attitude toward diabetes management is essential for adherence to treatment regimens and lifestyle changes, which directly influence QoL outcomes (11, 12).

In developing countries like Tanzania, the burden of non-communicable diseases, including type 2 diabetes, is rising rapidly. The Mwanza region, in particular, is experiencing an increase in diabetes prevalence, posing a significant public health challenge. Despite this, there is limited research on the knowledge, attitudes, and QoL among diabetic patients in this region. Understanding these factors is crucial for designing targeted interventions that can improve diabetes management and patient outcomes (13).

Research conducted in various parts of the world has highlighted the importance of diabetes education and its impact on patients’ QoL. For instance, studies in Saudi Arabia and Turkey have shown that educational interventions can lead to significant improvements in patients’ knowledge and attitudes toward diabetes, which in turn enhance their QoL (11, 14, 15). However, similar studies in the context of newly diagnosed diabetic patients in Mwanza, Tanzania, are lacking. This gap in knowledge underscores the need for research that specifically addresses the unique challenges faced by patients in this region (13, 16).

This study is guided by the health belief model (HBM), which suggests that individuals’ engagement in health-promoting behaviors depends on their perceived susceptibility, severity, benefits, barriers, and cues to action. This framework supports understanding the link between diabetes knowledge, attitudes, and quality of life.

## 2 Methodology

### 2.1 Study area

This study was conducted at the diabetic clinics of Bugando Medical Centre (BMC) in Mwanza, Tanzania, from September 2024 to December 2024. BMC is one of the largest tertiary hospitals in the Lake Zone of Tanzania, providing specialized medical services to a large population. BMC has a 1,000-bed capacity and serves as the zonal referral hospital for the Lake Zone, serving eight regions: Mwanza, Kagera, Kigoma, Mara, Geita, Shinyanga, Simiyu, and Tabora.

### 2.2 Study design

This study utilized a cross-sectional observational design.

### 2.3 Study population

The diabetic clinics at BMC cater to a significant number of patients with type 2 diabetes mellitus (T2DM), making it an ideal setting for assessing the knowledge, attitude, and QoL of newly diagnosed T2DM patients. The medical outpatient at this clinic attends to 100–130 patients daily from Monday to Friday every week, and among these patients, approximately 33 are diabetic.

### 2.4 Sample size estimation, selection criteria, and sampling method

The sample size was calculated using the Kish–Leslie formula, with a confidence interval of 95%, a marginal error of 5%, and an estimated prevalence of 0.242 of newly diagnosed T2DM patients with adequate knowledge, positive attitudes, or good QoL in the Malawian population (17). The minimum sample size was determined to be 130. Participants were selected using a serial convenience sampling method from the diabetic clinics at BMC, due to feasibility in a clinical setting, but this may limit generalizability. The inclusion criteria included newly diagnosed T2DM patients (diagnosed within the past 6 months), those aged 18 years and above, those attending diabetic clinics at BMC during the study period, and those who provided informed consent to participate. Patients with cognitive impairments or severe comorbidities that could hinder their ability to participate were excluded from the study.

### 2.5 Data collection and analysis

Data were collected using a structured questionnaire administered through face-to-face interviews. The questionnaire consisted of four sections: demographic information and clinical characteristics; knowledge assessment, which involved questions related to diabetes management, symptoms, complications, and preventive measures, using the Michigan Diabetic Knowledge Test (MDKT), with scores 0–9 indicating low knowledge and 10–14 indicating high knowledge; attitude assessment, which evaluated patients’ beliefs, perceptions, and attitudes toward diabetes and its management using the Diabetes Attitude Scale (DAS); and quality of life assessment using the WHOQOL-BREF questionnaire, with high (≥4), moderate (3 to 4), and low domain scores (<3) across four domains, namely, physical health, psychological health, social relationships, and environmental health, with overall quality of life categorized as follows: 0.0–3.9, poor QoL; 4.0–5.9, moderate QoL; 6.0–7.9, good QoL; and 8.0–10.0, very good QoL (17).

Data were analyzed using the SPSS (Statistical Package for the Social Sciences) software. Descriptive statistics (mean, standard deviation, frequencies, and percentages) was used to summarize the demographic and clinical characteristics, knowledge, attitudes, and QoL scores. Inferential statistics, including chi-square tests, *t*-tests, and regression analysis, was employed to identify associations between demographic variables and the main study outcomes (knowledge, attitudes, and QoL).

In addition to descriptive statistics, we performed bivariate analyses using chi-square and *t*-tests to explore associations between demographic/clinical variables and the main outcomes (knowledge, attitudes, and QoL). Due to sample size limitations, multivariate regression analysis was not fully implemented to control for potential confounders; however, we acknowledge that variables such as gender, education level, and comorbidities may have influenced the outcomes. In future studies with larger samples, regression modeling will be employed to adjust for these confounding factors and provide more robust inference.

### 2.6 Ethics

Ethical approval was sought from the Joint BMC/CUHAS Research Ethical Committee and Director of Research and Innovation of CUHAS (number CRECU/3246/2024). Written informed consent was sought and obtained before the recruitment of the study respondents, after they were provided with sufficient information about the risks and benefits of the study. The tools (WHOQOL-BREF and DAS) were administered in Swahili. Validated Swahili versions were used, following forward–backward translation protocols. Confidentiality was ensured, and those who agreed to participate signed the consent form, while illiterate participants provided a thumbprint.

## 3 Results

### 3.1 Sociodemographic characteristics

In this study, the median age of the newly diagnosed type 2 diabetic patients was 62 years (IQR 57–68). The majority of the participants were women (63.3%), while men constituted 36.7%. Most patients were married (92%), with only 8% in other marital statuses. Regarding education, 49.7% had primary education, 36.7% had secondary education, and 9.3% had tertiary education, while 4.7% had no formal education. A large proportion of the patients (82%) resided in urban areas, and only 18% lived in rural areas. Employment status showed that 35.3% were self-employed, 30% employed, 25.3% retired, and 9.3% unemployed. **Table 1** shows the sociodemographic information of the participants.

**Table 1:** Sociodemographic data of the participants (N=150).

Variable	Frequency, *N* (%)
Age, median (IQR)	62 years (57–68)
Gender	
Male	55 (36.7)
Female	95 (63.3)
Marital status	
Married	139 (92.7)
Others	11 (7.3)
Education level	
Non-formal	7 (4.7)
Primary	74 (49.7)
Secondary	55 (36.7)
Tertiary	14 (9.3)
Residence	
Rural	27 (18)
Urban	123 (82)
Employment	
Employed	45 (30)
Self-employed	53 (35.3)
Unemployed	14 (9.3)
Retired	38 (25.3)
Comorbid conditions	
None	38 (25.3)
Hypertension	110 (73.3)
Chronic illness	2 (1.3)
BMI, median (IQR)	28.4 kg/m^2^ (25–32.3)
SBP, median (IQR)	122 mmHg (107–135)
DBP, median (IQR)	79 mmHg (71–92)
HBA1C, median (IQR)	7.4% (6.9–8.8)

### 3.2 Clinical and laboratory characteristics

Among the patients, 66% had hypertension, while 18.7% reported no comorbid conditions and 15.3% had other chronic illnesses. The median BMI was 28.4 kg/m² (IQR 25–32.3), indicating that many patients were overweight or obese. The median systolic blood pressure (SBP) was 122 mmHg (IQR 107–135), and the median diastolic blood pressure (DBP) was 79 mmHg (IQR 71–92). The median HbA1c level was 7.4% (IQR 6.9–8.8) (**Table 1**).

### 3.3 Knowledge assessment

Among the 150 participants, the results indicate that majority (70.6%) of the participants had low knowledge of diabetes, with a median score of 9 (IQR 7–10). Gender-wise, 63.6% of men and 74.7% of women had low knowledge, showing a slightly lower knowledge level among women. Education level played a significant role, as participants with tertiary education had the highest knowledge (57.1%), while those with primary education had the lowest (21.6%) (*P*-value=0.05). Residence had a little impact, with 72.4% of urban participants and 63% of rural participants having low knowledge. Employment status also influenced knowledge, with 85.7% of unemployed participants having low knowledge compared to 80% of employed and 62.3% of self-employed individuals (*P*-value=0.13). Regarding comorbidities, 79.3% of those without any condition and 68.2% of hypertensive patients had low knowledge (*P*=0.42). These findings highlight the urgent need for targeted educational interventions, particularly for women, individuals with lower education levels, the unemployed, and those living in urban areas, to enhance diabetes awareness and management (**Tables 2**, **3**).

**Table 2:** Overall knowledge of the participants (N=150).

Variable	Frequency, N (%)
Median score 9 (IQR 7–10)	(7–10)
Low	106 (70.6)
High	44 (29.4)
Total	150 (100)

**Table 3:** Knowledge of the participants (N=150).

Variable	Frequency, N (%)	Knowledge, N (%)		P-value
Gender		Low	High	
Male	55 (36.7)	35 (63.6)	20 (36.4)	
Female	95 (63.3)	71 (74.7)	24 (25.3)	0.15
Marital status				
Married	139 (92.7)	99 (71.2)	40 (28.8)	
Others	11 (7.3)	7 (63.6)	4 (36.4)	0.6
Education level				
Non-formal	7 (4.7)	4 (57.1)	3 (42.9)	
Primary	74 (49.7)	58 (78.4)	16 (21.6)	
Secondary	55 (36.7)	38 (69.1)	17 (30.9)	0.05
Tertiary	14 (9.3)	6 (42.9)	8 (57.1)	
Residence				
Rural	27 (18)	17 (63)	10 (37)	
Urban	123 (82)	89 (72.4)	34 (27.6)	0.33
Employment				
Employed	45 (30)	36 (80)	9 (20)	
Self-employed	53 (35.3)	33 (62.3)	20 (37.7)	0.13
Unemployed	14 (9.3)	12 (85.7)	2 (14.3)	
Retired	38 (25.3)	25 (65.8)	13 (34.2)	
Comorbid conditions				
None	38 (25.3)	29 (79.3)	9 (23.7)	
Hypertension	110 (73.3)	75 (68.2)	35 (31.8)	0.42
Chronic illness	2 (1.3)	2 (100)	0 (0)	

*Percentages may not total 100% due to rounding.

### 3.4 Attitude assessment

In the attitude assessment of newly diagnosed type 2 diabetic patients, 54.9% exhibited a negative attitude toward their condition, while 45.1% had a positive attitude. Key indicators showed that 52.7% of patients were afraid of their diabetes, and 49.3% found it hard to believe they had the disease. Additionally, 61.3% felt inferior due to their condition, and 64% found it difficult to manage the necessary daily tasks. On the positive side, 50% felt things were going well in their lives, and 48% believed diabetes did not significantly affect their lives (**Table 4**).

**Table 4:** Attitude assessment of the participants (N=150).

SN	Parameter	Negative attitude, frequency (%)	Positive attitude, frequency (%)
1	I am afraid of my diabetes	79 (52.7)	71 (47.3)
2	I find it hard to believe that I really have diabetes	74 (49.3)	76 (50.7)
3	I feel unhappy and depressed because of my diabetes	79 (52.7)	71 (47.3)
4	I feel satisfied with my life	93 (62)	57 (38)
5	I feel I am not as good as others are because of my diabetes	92 (61.3)	58 (38.7)
6	I can do just about anything I set out to do	78 (52)	72 (48)
7	I find it hard to do all the things I have to do for my diabetes	96 (64)	54 (36)
8	Diabetes does not affect my life at all	78 (52)	72 (48)
9	I am pretty well off, all things considered	79 (52.7)	71 (47.3)
10	Things are going very well for me right now	75 (50)	75 (50)

*Percentages may not total 100% due to rounding.

### 3.5 Quality of life assessment

The assessment of the quality of life among newly diagnosed type 2 diabetic patients at BMC revealed varying levels of wellbeing across multiple domains. The physical health domain had a mean score of 3.1 (SD ± 0.56) and the psychological domain had a slightly higher mean score of 3.2 (SD ± 0.61), both indicating average quality of life. The social domain showed a median score of 3.7 (IQR 2.7–3.7), suggesting that patients generally perceived their social interactions positively. However, the environmental domain score was lower, with a mean of 2.99 (SD ± 0.53), highlighting challenges in living conditions and access to healthcare. The median score of overall quality of life was 3.2 (IQR 2.8–3.5), indicating a need for targeted interventions to improve quality of life, particularly in environmental factors, to support better diabetes management and enhance patients’ daily experiences (**Table 5**).

**Table 5:** Quality of life assessment of the participants (N=150).

Variable	Frequency, N (%)	
Gender	Poor QoL	Good QoL
Male	52 (36.6)	3 (37.5)
Female	90 (63.4)	5 (62.5)
Domain	Mean/median	
Physical health	Mean 3.1 (SD ± 0.56)	
Psychological health	Mean 3.2 (SD ± 0.61)	
Social relationship health	Median 3.7 (IQR 2.7–3.7)	
Environmental health	Mean 2.99 (SD ± 0.53)	
Overall quality of life	Median score 3.2 (IQR 2.8–3.5)	

## 4 Discussion

### 4.1 Sociodemographic characteristics

This study found that the median age of newly diagnosed type 2 diabetic patients was 62 years (IQR 57–68), and the majority of these patients were women (63.3%). In contrast, some studies with larger sample sizes have reported younger median ages for type 2 diabetes diagnosis, and their gender distribution varies. In another study, the mean age was slightly younger, and the gender distribution was more balanced between men and women (17, 18). These differences could be attributed to variations in regional health profiles, where certain populations may experience an earlier onset of diabetes or a more even gender distribution.

In terms of education level, 49.7% of the participants in this study had primary education, which is somewhat higher compared to the 36.7% observed in a study conducted, where a large proportion of participants had secondary education (11). The differing education levels between these studies may be due to regional disparities in educational access and socioeconomic status, which can influence health outcomes and disease management.

### 4.2 Clinical and laboratory characteristics

The prevalence of hypertension among participants in this study of 66% was consistent with other studies; however, this percentage was higher than what was reported in some studies (46%) (19). The variation in hypertension rates could be attributed to different healthcare environments, diagnostic criteria, and lifestyle factors prevalent in different regions. A study found that hypertension rates were lower in their cohort, potentially due to differences in health awareness and preventive measures available in those regions (19).

Additionally, the median HbA1c level of 7.4% (IQR 6.9–8.8) in this study suggested suboptimal glycemic control, which is comparable to the findings of another study, where HbA1c levels were similarly elevated (11, 18). However, other studies reported significantly better glycemic control, with median HbA1c values ranging between 6.5% and 6.8% (20). The observed differences may be due to variations in patient adherence to treatment regimens, healthcare access, or cultural differences in diabetes management practices.

### 4.3 Knowledge assessment

The findings of this study indicated that the overall knowledge of diabetes among newly diagnosed type 2 diabetic patients at Bugando Medical Centre was generally low, with 70.6% of participants demonstrating inadequate knowledge. This aligns with the findings from another study conducted in a rural community in the Philippines, where a significant proportion of diabetic patients exhibited poor understanding of diabetes management, highlighting the need for targeted educational interventions (11). Similarly, research in Singapore established a strong association between diabetes knowledge and health-related quality of life (HRQoL), suggesting that inadequate knowledge contributes to poor disease management and overall wellbeing (2). In contrast, studies have shown that structured self-management training significantly improves diabetes knowledge and enhances patient outcomes (21). The present study also found variations in knowledge levels based on demographic factors, with male participants showing slightly better knowledge (36.4%) compared to women (25.3%). Education level was a significant determinant, with participants who attained tertiary education displaying the highest proportion of adequate knowledge (57.1%), whereas those with primary education had the lowest (21.6%). These findings emphasize the critical role of education in diabetes management and reinforce previous research indicating that higher educational attainment correlates with improved disease understanding and self-care practices (21). Moreover, knowledge disparities between urban and rural residents, as well as among different employment categories, suggest that socioeconomic factors may influence access to diabetes-related information, further reinforcing the need for comprehensive educational programs tailored to diverse patient populations.

### 4.4 Attitude assessment

The assessment of attitudes toward diabetes revealed a concerning prevalence of negative attitudes, with over half (54.9%) of the participants expressing fear and dissatisfaction regarding their condition. Many patients reported feelings of unhappiness and depression associated with their diabetes diagnosis, with 52.7% indicating that they feel unhappy and 61.3% feeling inferior due to their condition. Similar findings have been reported in other studies, where negative attitudes were associated with poor adherence to treatment regimens (17, 22). Positive attitudes, reported by 45.1% of participants, indicated a subset of patients who view their condition as manageable, highlighting the potential of psychosocial interventions (12, 14). However, the study, which had a larger cohort, found a higher proportion of patients exhibiting positive attitudes (58%) (18). This difference could be due to regional cultural factors that influence how diabetes is perceived, as well as differences in the healthcare support systems available to patients. In contrast, studies found lower levels of negative attitudes (approximately 40%), likely due to different healthcare systems, educational outreach, and societal norms around chronic illness (11, 20). Notably, addressing psychological barriers through counseling and support groups can significantly improve patients’ attitudes toward their health, suggesting that a multifaceted approach is needed to enhance both knowledge and psychological wellbeing among diabetic patients. These findings highlight the necessity for healthcare providers to address not only the educational needs of patients but also their emotional and psychological needs, ensuring that patients feel supported throughout their diabetes journey.

### 4.5 Quality of life assessment

The assessment of QoL indicated that a significant proportion of patients reported poor quality of life, particularly in the physical and environmental domains. The mean scores were 3.1 (SD ± 0.56) for physical health and 2.99 (SD ± 0.53) for environmental quality, suggesting that many patients struggle with the physical aspects of their condition and face challenges in their living environments. These findings align with other studies, where environmental factors such as access to healthcare and living conditions were key determinants of HRQoL (18, 20). The psychological domain mean score of 3.2 reflects moderate mental health challenges, consistent with global data emphasizing the impact of diabetes on psychological wellbeing (12, 17). The social domain showed a relatively higher median score of 3.7, suggesting that social support systems might mitigate some of the disease burden (11). However, the study reported significantly higher scores in environmental health (mean 3.6) (18). This difference could be due to variations in healthcare infrastructure and the availability of diabetes care resources. The lower environmental domain score in this study may indicate a greater need for targeted interventions to improve the living conditions of diabetic patients, particularly in regions with limited healthcare access.

The overall quality of life median score of 3.2 (IQR 2.8–3.5) in this study is similar to the findings of another study, where quality of life scores were also moderate (mean 3.0) (11). However, studies with larger and more diverse populations reported somewhat higher quality of life scores (mean 3.5–4.0) (18, 20). This variation could reflect differences in healthcare delivery systems, cultural perceptions of diabetes, and patient access to psychosocial support, all of which contribute to overall wellbeing.

It was also observed that women reported a poorer quality of life compared to men. Specifically, 90 out of 95 women (94.7%) were categorized as having poor quality of life, while only 52 out of 55 men (94.5%) fell into this category. In a previous study, women reported poorer quality of life in several domains, including physical and psychological health, compared to men (18). The findings suggest that women may experience a more significant impact of diabetes on their daily lives, potentially due to additional social and psychological factors. This emphasizes the importance of considering gender as a potential determinant of health outcomes and highlights the need for tailored interventions for women with diabetes in some settings. Addressing these gender-specific challenges through targeted interventions is critical to improving health outcomes for both genders. Programs that consider the unique circumstances of female patients, including family support and access to healthcare, can help enhance their quality of life and promote better diabetes management.

### 4.6 Implications for healthcare

The findings of this study underscore the urgent need for enhanced diabetes education and support services tailored to the unique needs of newly diagnosed patients. Integrating educational programs that address both knowledge gaps and psychological barriers can empower patients to manage their diabetes effectively. This indicates that patient education programs can improve self-management and enhance patients’ confidence in managing their condition. Collaborative care approaches, involving multidisciplinary teams, can provide comprehensive support that encompasses medical treatment, psychological counseling, and lifestyle modifications. Additionally, incorporating community resources and support groups can help create a more supportive environment for patients managing diabetes.

Moreover, healthcare providers should consider implementing routine assessments of patients’ knowledge, attitudes, and quality of life as part of standard care. Regular follow-ups can help identify patients at risk of poor outcomes and provide targeted interventions to address their specific needs. Training healthcare providers to recognize and address psychological issues related to diabetes management is also crucial in promoting a more holistic approach to care. By fostering an environment that encourages open communication and support, healthcare professionals can help patients navigate their diabetes journey more effectively.

### 4.7 Study limitations

The use of specific quality of life assessment tools may not fully capture all dimensions of patients’ experiences. Although differences were observed across gender and education levels, these associations were not adjusted for confounders due to sample size constraints. This limits the strength of conclusions regarding these variables. This study employed serial convenience sampling, which may introduce selection bias. Patients who attend clinics more regularly or those more willing to participate may differ systematically in their knowledge, attitudes, or QoL compared to non-attendees or those who declined participation. As a result, the findings may overrepresent individuals who are more health-conscious or have better healthcare access, limiting the generalizability of our results to the broader diabetic population at BMC and similar settings.

### 4.8 Recommendations

To improve the management and quality of life for newly diagnosed type 2 diabetic patients, it is crucial to implement targeted educational programs that enhance diabetes knowledge, particularly for vulnerable groups such as female patients. Adopting a multidisciplinary care approach can ensure that patients receive comprehensive support tailored to their needs. Regular assessments of knowledge, attitudes, and quality of life should be integrated into standard care practices, allowing for timely interventions. These programs should be complemented by psychological support services that address the emotional challenges associated with the disease. Finally, fostering community engagement and developing gender-sensitive strategies will further support patients in managing their diabetes effectively, ultimately leading to improved health outcomes.

### 4.9 Conclusion

This study underscores the significant challenges faced by newly diagnosed type 2 diabetic patients at Bugando Medical Centre in Mwanza, Tanzania, particularly concerning knowledge gaps, negative attitudes, and diminished quality of life. With a moderate knowledge score and a high prevalence of negative perceptions about diabetes, it is evident that many patients are ill-prepared to manage their condition effectively. These findings emphasize the need for comprehensive educational initiatives and psychological support to empower patients in their self-management efforts. Furthermore, the reported poor quality of life, particularly in the physical and environmental domains, highlights the urgency for healthcare interventions, such as developing structured diabetes education sessions tailored to the patients’ literacy level, training nurses and community health workers in psychosocial support for newly diagnosed patients, and integrating routine QoL and attitude assessments into diabetic clinic visits, which address both the clinical and psychosocial aspects of diabetes care.

## Data Availability

The original contributions presented in the study are included in the article/supplementary material. Further inquiries can be directed to the corresponding author.
